# A Traditional Chinese Medicine for the Treatment of Endometrial Hyperplasia via Regulating the HPO Axis in Rats

**DOI:** 10.1155/2022/5200608

**Published:** 2022-02-02

**Authors:** Bo Lv, Yuan-de Peng, Wen-hui Fu, Wen-jing Li, Li-jun Chen, Zhi Wang

**Affiliations:** ^1^Key Laboratory of Protein Chemistry and Developmental Biology of Fish of Ministry of Education, Hunan Normal University, Changsha 410081, China; ^2^Institute of Bast Fiber Crops, Chinese Academy of Agricultural Sciences, Changsha 410205, Hunan, China; ^3^College of Bioscience and Biotechnology, Hunan Agriculture University, Changsha 410128, Hunan, China; ^4^Shaoyang University, Shaoyang 422000, Hunan, China

## Abstract

Dysfunctional uterine bleeding, accompanied by endometrial hyperplasia (EH), is a common gynecological disease that seriously affects female physical and mental health. Some drugs have been prompted to cure the disease, but most medications have certain side effects and limitations. In the present study, we demonstrated an unexploited Chinese traditional medicine, a combination of *Saururus chinensis*, *Celosia cristata*, and *Spatholobus suberectus* (SCS), which could be used for the treatment of EH and associated complications in rats. We identified the active components from the three Chinese herbs via thin-layer chromatography and high-performance liquid chromatography methods. In addition, serum biochemical indexes and histologic section results found that acute high-dose SCS exerted no adverse impacts on the rats. We then showed that SCS shortened coagulation time (*p*=0.018) and degree of swelling (*p*=0.021) on rats at 30 min compared to blank control. Further studies proved that recovered endometrial thickness was associated with the modulation of four hormones (follicle-stimulating hormone, luteinizing hormone, estrogen, and progesterone). Specifically, follicle-stimulating hormone and progesterone contents increased gradually with time, and estrogen was decreased, whereas luteinizing hormone content was returned to normal after a short-term elevation (*p* < 0.05). Besides, SCS increased uterine endometrium's mRNA expression levels of matrix metalloproteinase-1 (*p* < 0.001) and tissue inhibitor of matrix metalloproteinase-1 (*p* < 0.001), promoting the repair of proliferating endometrium in the rats. Collectively, our study indicates that SCS harbors a profoundly curative effect on the treatment of EH and relative complications and uncovers the mechanism at molecular and gene expression levels.

## 1. Introduction

Dysfunctional uterine bleeding (DUB), accompanied by proliferating endometrium, which is characterized as excessively heavy, prolonged, and frequent bleeding of uterine origin, is a common disease to the Emergency and Gynecology Department [1]. The earliest endometrial hyperplasia (EH) characterized by crowded glands with simple tubular structure lined by cells resembling proliferative endometrium and a mushroom-like membrane projection (pinopode) is the critical indicator of endometrial receptivity [2, 3]. The primary cause of EH and DUB is the dysfunction of the HPO (hypothalamus-pituitary-ovarian) axis [4], which is related to the expression of female hormones, including follicle-stimulating hormone (FSH), luteinizing hormone (LH), progesterone, and estrogen [5, 6]. The sudden decrease of estrogen in proliferating endometrium patients induces apoptosis of epithelial cells of endometrial glands, resulting in irregular shedding of the endometrium [1]. Also, the extracellular matrix of endometrium contains a variety of macromolecules such as collagen, glycoprotein, and proteoglycan, which are regulated by two key enzymes, matrix metalloproteinase (MMP) and tissue inhibitor of matrix metalloproteinase (TIMP) [7]. TCM treatment studies have shown promising results for delayed menstrual cycles to cure the DUB and proliferating endometrium [8]. Furthermore, most standard treatments are given priority to oral sex hormone drugs and injections, such as nonsteroidal anti-inflammatory drugs, tranexamic acid, prostaglandins, and estrogen [9, 10]. However, most drugs have certain side effects, which are accompanied by nausea, vomiting, and headaches [11–13]. Hence, it is essential to promote the development of new effective drugs for the treatment of EH and DUB patients.

Traditional Chinese medicine (TCM) is well documented to play a role in protecting and curing fertility-related diseases. Nonetheless, scientifically proving the efficacy of TCM, such as the primary active components and cute high-dose toxicity, is necessary. Particularly, the standards of TCM on fertility factors, like ovulation rates, female hormone, and appropriate thickness of the endometrial lining, are essential biomarkers for the assessment of TCM on fertility-related diseases. Several drugs and extractions have been demonstrated to play a key role in strengthening coagulation, reducing inflammation, and lowering uterine bleeding in the patients. *Saururus chinensis*, a perennial herb widely distributed in northeastern Asia, is able to fight against inflammation, oxidative stress, and proliferation, which permit to treat hyperplasia [14–17]. Similarly, *Celosia cristata* is applied for the treatment of painful menstruation, abdominal pain, and hemoptysis [18–20]. In addition, evidence has demonstrated that *Spatholobus suberectus* exhibits various functions against oxidation, viruses, bacteria, cancer, and platelets [21–23]. Here, we identified that a combination of *Saururus chinensis*, *Celosia cristata*, and *Spatholobus suberectus* (SCS) possesses positive effects on the treatment of EH and relative complications. Briefly, we found that SCS shortened coagulation time and alleviated proliferative endometrium via the regulation of four hormones and two key genes involved in the HPO axis.

## 2. Materials and Methods

### 2.1. Drugs Preparation

*S. chinensis*, *C. cristata*, and *S. suberectus* decoction pieces were purchased from Hunan Gaoqiao Market (Changsha, China), and the standard control decoction pieces were obtained from China Institute for the Inspection of Medicines and Biological Products to identify the components in the SCS decoction pieces. Each medicinal material was made into coarse powder by an ordinary pulverizer and then pulverized by an ultrafine pulverizer after oven-drying (60°C, 12 h). The obtained medicinal ultrafine pieces were stored in a ventilated and dry place for later use.

### 2.2. Active Components Identification by TLC

*S. chinensis* test solution (10 *µ*L) includes roots, stems, leaves, and standard control medicinal solution (10 *µ*L), which were, respectively, added into a silica gel plate and then placed in a chromatographic cylinder containing 10 mL petroleum ether (60～90°C) and acetone. Finally, the dried samples were sprayed with 10% sulfuric acid ethanol coloring solution and heated at 105°C until showing clear spots.

*C. cristata* test solution (10 *µ*L) and standard control medicinal solution (10 *µ*L) were added into a silica gel plate and then placed in a chromatographic cylinder containing 10 mL cyclohexane-acetone (5 : 1) separately. The two samples were colored by 5% vanillin sulfuric acid and heated at 105°C until showing clear spots.

*S. suberectus* test solution (10 *µ*L) and standard control medicinal solution (10 *µ*L) were, respectively, added into the silica gel plate and then placed in a chromatographic cylinder containing 10 mL chloroform-methanol (20 : 1). The dried samples were observed at 254 nm in the ultraviolet gel imaging system and photographed.

### 2.3. Quantitative Determination of Active Components by HPLC

To evaluate the concentration of obtained decoction pieces, we used the HPLC method to test the quality of *S. chinensis* and *S. suberectus* according to the *Chinese Pharmacopoeia 2010* [24] (standard sample for *C. cristata* was not recorded). A total of 0.5 g *S. chinensis* was added into 25 mL methanol for 30 min and then treated with ultrasound (500 W, 25 kHz) for 40 min. The standard sample sauchinone was taken and made into a 40 ug/mL solution with methanol. The two types of samples were then detected by an HPLC (YOUDAO2000). The previous treatment of *S. suberectus* was consistent with *S. chinensis*. The standard control sample formononetin was added into a 40 ug/mL solution with methanol. Again, the two types of samples (formononetin and *S. suberectus*) were detected by the HPLC.

### 2.4. Animals and Treatment

According to the conversion reaction on the basis of *Chinese Pharmacopoeia* [24], three normal-dose herbs weighing 0.875 g (i.e., *S. chinensis* weighed 0.375 g, *C. cristata* weighed 0.25 g, and *S. suberectus* weighed 0.25 g) were added into 1 L 100% ethanol and boiled for 30 min. Similarly, the 20-dose group weighed a total of 17.5 g herbs. After cooling, the residue was filtered and added with 1 L distilled water boiling for 30 min, and the residue was filtered again. The obtained alcohol extract was concentrated into powder in a vacuum freeze dryer and stored for later use. Before intragastric administration, the yielded extract was dissolved with a proper amount of saline and reserved at 4°C.

Female SD rats purchased from the Hunan SJA Laboratory Animal Co., Ltd. (Changsha, China) were used in all experiments. The SD rats were kept in specific pathogen-free conditions in the animal experiment centre of Hunan Normal University. The diets of the rats were provided with Grade B rat breeding stocks daily, and the rats had ad libitum access to food and water during the study. All of the protocols for this study were approved by the Ethics Committee of Hunan Normal University. The procedures of this study were performed in accordance with the guidelines of the European Community (directive 2010/63/EU) for the care and use of experimental animals.

### 2.5. Toxicity Evaluation of Acute High-Dose SCS

A total of 12 healthy SD female rats, weighing 130 ± 20 g, were habitually reared for one week and randomly divided into the following three groups: blank control group (CK, intragastrically administered equal volume of normal saline), normal dose SCS administration group (0.875 g/kg/d, SCS), and 20-dose SCS administration group (17.5 g/kg/d, SCS 20×). The normal dose and 20-dose SCS solutions were obtained as previously described in Section 2.4 and used for administration, respectively. Four SD female rats per group were intragastrically given the corresponding solution concentration at a fixed time for seven consecutive days and fed with 25 g/d Grade B rat breeding stocks. The rats were sacrificed under light ether anesthesia at the end of the treatment and the plasma samples were collected to calculate visceral coefficients and blood biochemical parameters.

In addition, to assess the safety of SCS on the rats, we collected two organs (liver and kidney) from the rats and depicted histologic sections based on the samples. The clean organ samples were immersed in neutral formalin for one week and then dehydrated with a gradient of ethanol. The xylene solution was used for transparent treatment, and then the samples were embedded with paraffin. Paraffin sections were sectioned at 5 *μ*m thickness and mounted on superfrost plus slides. Every tenth section was stained with hematoxylin and eosin and examined by light microscopy.

### 2.6. Determination of Hemostasis and Coagulation Effects

Healthy SD female rats, weighing 200 ± 20 g, were adaptively reared for one week and randomly divided into the following three groups (each group had four rats): CK (intragastrically given normal saline), normal dose Gongxuening positive control group (a certified Chinese patent medicine with curative effects on DUB, 0.13 g/kg/d, GXN) [25], and normal dose SCS group (SCS). Four female rats per cage were intragastrically given at a fixed time every day for 30 d. Thirty minutes after the end of gavage on the 30^th^ d, the tail was cut off 0.5 cm from the tip of the rat, and the time was counted from the blood flowed out spontaneously to stop bleeding. Meanwhile, we used a clean pin every 15 s to pick up the bottom of the blood drop until the fibrin filaments showed up and then recorded the clotting time. Furthermore, each rat's right foot was injected with 0.1 mL 10% egg white saline. The foot volume of the rats was recorded at 10 min, 20 min, and 30 min after injection, and the foot swelling volume data were obtained to calculate the detumescent degree of rats.

### 2.7. Endometrial Hyperplasia Rat Establishment

Healthy SD female rats, weighing 200 ± 20 g, were reared adaptively for one week and randomly divided into blank control (CK, *n* = 8) and model group (*n* = 24). The rats in the model group were intramuscularly injected with estradiol benzoate 1 mg/kg every other day to yield EH model rats as previously described with minor modifications [26]. After 60 d injections, the EH model rats were randomly divided into three groups: EH model group (EH, gavage of equal volume of normal saline), normal dose GXN group (GXN, 0.13 g/kg/d), and SCS group (SCS, 0.875 g/kg/d). The control group was fed the same amount of vehicle (dimethyl sulfoxide). The two different drugs (GXN and SCS) were uniformly prepared with saline for suspension, and the CK group was intramuscularly injected with 0.9% sodium chloride injection. On the 0 d, the 3 d, and the 7 d of administration, four rats were randomly selected from each group to test the thickness of the endometrium and serous hormone activity.

### 2.8. Scanning Electron Microscopy

The histological alterations in the endometrium were detected by scanning electron microscopy (SEM). After anesthetization, an incision (3 cm) was cut at the lower abdomen of rats and then an uterine horn (1 cm) was separated from the incision. The endometrium was separated with forceps and fixed with 2% pentanediol and 1% osmic acid, followed by dehydration with a gradient of ethanol. The samples were then embedded, and semithin sections and ultrathin sections were yielded by the microtome. The images were photographed under SEM (model Carl Zeiss-EVO-40) with the accelerated voltage of 10 kV at a magnification of ×2000.

### 2.9. Determination of Four Key Enzymes and Gene Expression of MMP-1 and TIMP-1

As previously mentioned [4], four crucial hormones regulated by the HPO axis are related to endometrial hyperplasia. Hence, the concentrations of progesterone, estrogen, FSH, and LH in the blood of SD rats (propstrum) were determined in accordance with the principles and procedures of ELISA (enzyme-linked immunosorbent assay) kits (Shanghai Jianglai Biological Co., Ltd.). The optical value of each sample was measured at 450 nm using an ELISA reader (Synergy HTX, BioTek).

The ovaries and uterus were taken immediately after dissection of rats, and the mRNA expression levels of matrix metalloproteinase-1 (MMP-1) and tissue inhibitor of matrix metalloproteinase-1 (TIMP-1) were detected by RT-qPCR technology. The Ace qPCRSYBR Green Master Mix (Vazyme, Nanjing, China) was used to transcribe the purified RNA from rat samples. Fluorescent primer design was performed using Primer 6.0, and the detailed sense primer sequences were “ACTCCCTTGGACTCACTCATT” and “ACCAGCGTTATGAGATCAAGATGA”, and the antisense primer sequences were “GTGTTGTTGCACCTGTTGG” and “TGCCCAGGGAACCAGGAA” for MMP-1 and TIMP-1, respectively. Actin-*β* gene was used as internal reference for RT-qPCR primer sequence. The amplification process was one cycle of 95°C (5 min), 40 cycles of 95°C (15 s), 55°C (30 s), and 72°C (30 s) and followed by a melt curve test using a temperature range from 60°C to 95°C. Three biological replicates were run in the RT-qPCR system. The 2^−ΔΔCT^ method was adopted to analyze the relative expression levels of genes.

### 2.10. Data Analysis

Statistical analysis related to the concentration of organ coefficient, serum biochemical index, and enzymes and gene expression levels was performed using one-way ANOVA analysis, followed by a post hoc test, or two-way repeated-measures ANOVA (GraphPad Prism, V.5.02, GraphPad Software, Inc.). Adobe Photoshop CS6 and Adobe Illustrator CC were utilized for figure processing. The values were presented as the mean ± standard error.

## 3. Results

### 3.1. Detection of Primary Functional Components of Medicinal Materials

**Figure 1:**
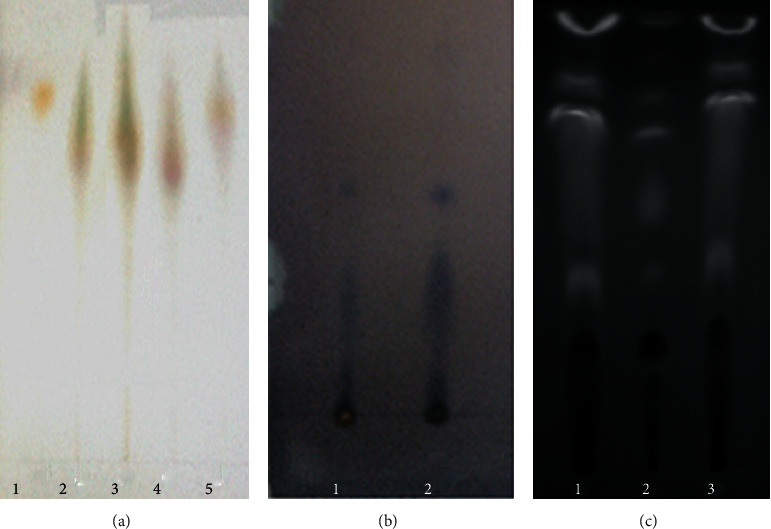
Thin-layer chromatography (TLC) images of three herbs. (a) TLC chromatogram of *S. chinensis* (1: *S. chinensis*, 2: control herb, 3: leaf, 4: root, 5: stem). (b) TLC chromatogram of *C. cristata* (1: control herb, 2: *C. cristata*). (c) TLC chromatogram of *S. suberectus* (1 and 3: control herb, 2: *S. suberectus*).

**Figure 2:**
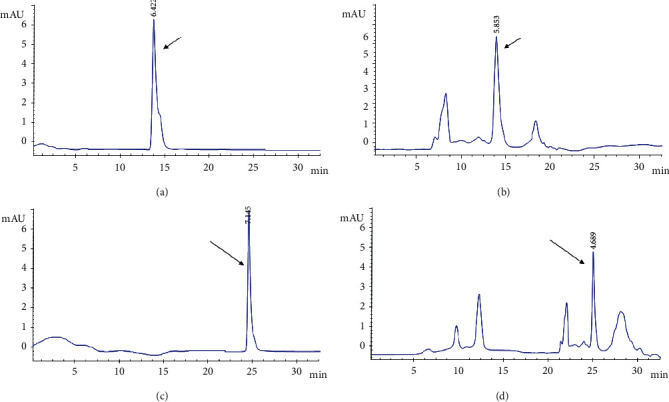
High-performance liquid chromatography (HPLC) images of *S. chinensis* and *S. suberectus*. (a) HPLC chromatogram of control herb (sauchinone). (b) HPLC chromatogram of *S. chinensis*. Arrow refers to the sauchinone. (c) HPLC chromatogram of control herb (formononetin). (d) HPLC chromatogram of *S. suberectus*. Arrow refers to the formononetin.

The present study identified the active components from three decoction pieces (*S. chinensis*, *C. cristata*, and *S. suberectus*) with relative standard drugs. As shown in Figure 1, TLC chromatography of *S. chinensis* leaves, roots, and stems suggested that the *S. chinensis* drug displayed the same color spots as the standard medicine of the control. Meanwhile, both *C. cristata* and *S. suberectus* presented similar spots on the corresponding positions of the standard control chromatography. In addition, the results of HPLC showed that *S. chinensis* and *S. suberectus* depicted the same expression pattern on the main functional peaks as the corresponding control drugs of sauchinone (*y* = 2.3904*x* + 0.435, *R*^2^ = 0.9999) and formononetin (*y* = 1.6514*x* + 0.411, *R*^2^ = 0.9997) (Figure 2), demonstrating that sauchinone and formononetin were two active substances in the SCS drug.

### 3.2. High-Dose SCS Exerted No Significant Toxic Effects on Rats

**Figure 3:**
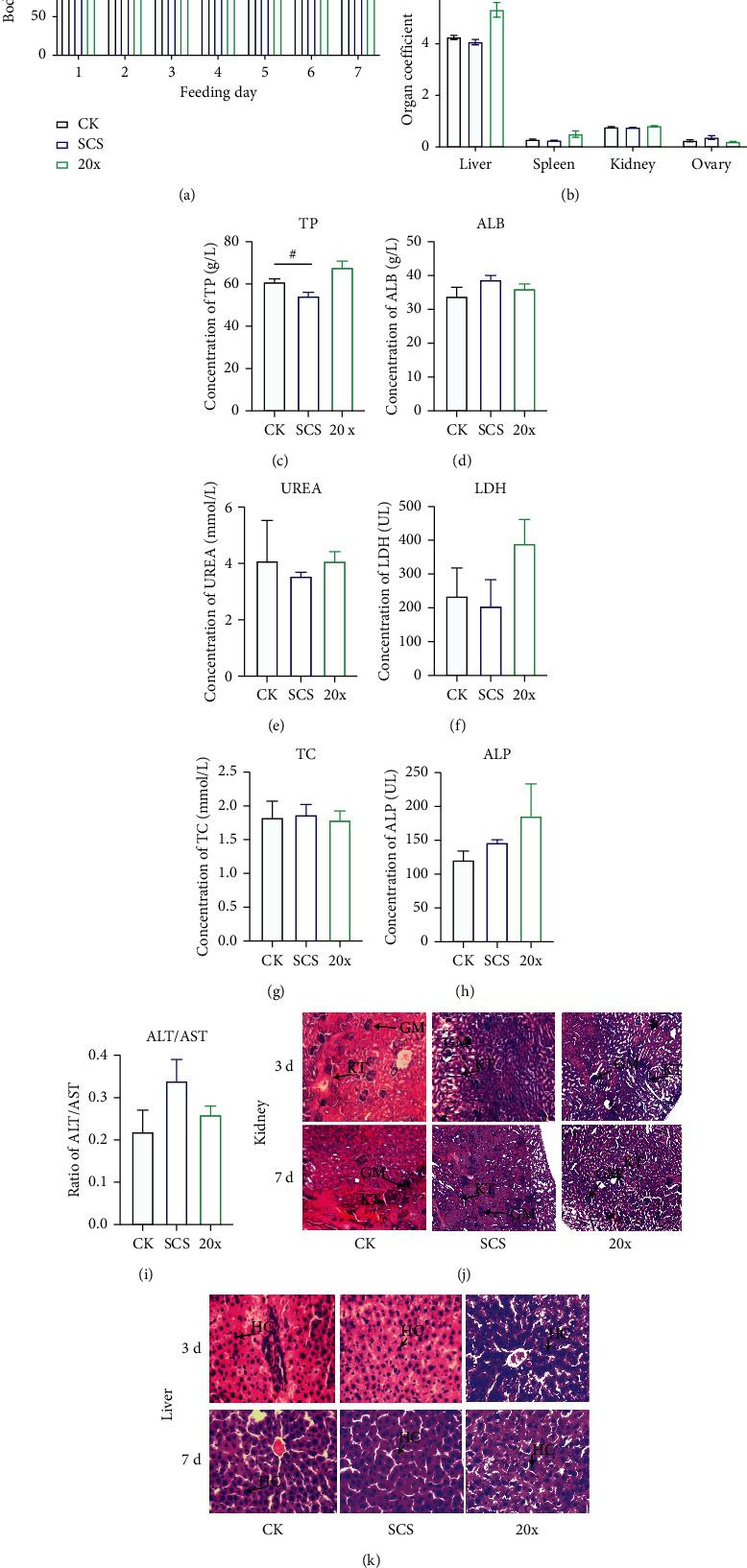
The effects of acute high-dose SCS drug on the SD rats. (a) Histogram of body mass alterations in high-dose SCS treated rats. (b) Effects of acute medication on the organ coefficients in rats. The organ coefficient is expressed by dividing the organ weight by body mass. (c–i) The alterations of blood biochemical parameters under the gavage of acute SCS. (j) Histologic section images of the rat kidney after 3 and 7 d of gavage. (k) Histologic section images of the rat liver after 3 and 7 d of gavage. ^*∗*^Asterisk (^*∗*^) indicates the significant difference (*p* < 0.05, *n* = 4). Abbreviations: GM, glomeruli; KT, kidney tubes; HC, hepatocyte; TP, total protein; ALB, albumin; TC, total cholesterol; ALT, alanine aminotransferase; AST, aspartate aminotransferase; LDH, lactic dehydrogenase; ALP, alkaline phosphatase.

The rats were closely observed after daily administration, and the results represented that no distinctive body weight alterations were found in the three comparisons (Figure 3(a)). Notably, the organ coefficient value of the spleen, kidney, and ovary displayed no changes (*p* > 0.05), but the index of the liver increased significantly (*p*=0.011) in the high-dose SCS treated rats (Figure 3(b)), whereas the normal dose SCS drug showed no liver coefficient alterations comparing with the CK group (*p*=0.194) (Figure 3(b)). According to the results of blood biochemical indexes, acute high-dose SCS drug treatment did not trigger adverse effects on rats. To be more specific, the concentration of total protein, albumin, urea, total cholesterol, lactic dehydrogenase, and alkaline phosphatase and the ratio of alanine aminotransferase/aspartate aminotransferase (ALT/AST) displayed no significant biological difference between acute high-dose SCS and control rats (*p* > 0.05, Figures 3(c)∼3(i)). Besides, the content of plasma total protein content was depleted in normal SCS-treated rats (*p*=0.021), but the value was within the normal range.

We subsequently demonstrated the safety of SCS according to the histologic section images of liver and kidney. The pathological section of the rat kidney showed that the numbers of glomeruli and tubules in the SCS normal and high-dose group were slightly higher than those of control rats but with no other significant alterations (Figure 3(j)). Liver pathological section results showed that gap junctions between the hepatocytes in the SCS (both normal and high-dose groups) groups and control group did not alter significantly on the 3 d and 7 d (Figure 3(k)), indicating that the SCS exerted no significant side effects in the kidney or liver.

### 3.3. SCS Drug Showed Hemostasis, Anti-Inflammation, and Detumescence Effects

**Figure 4:**
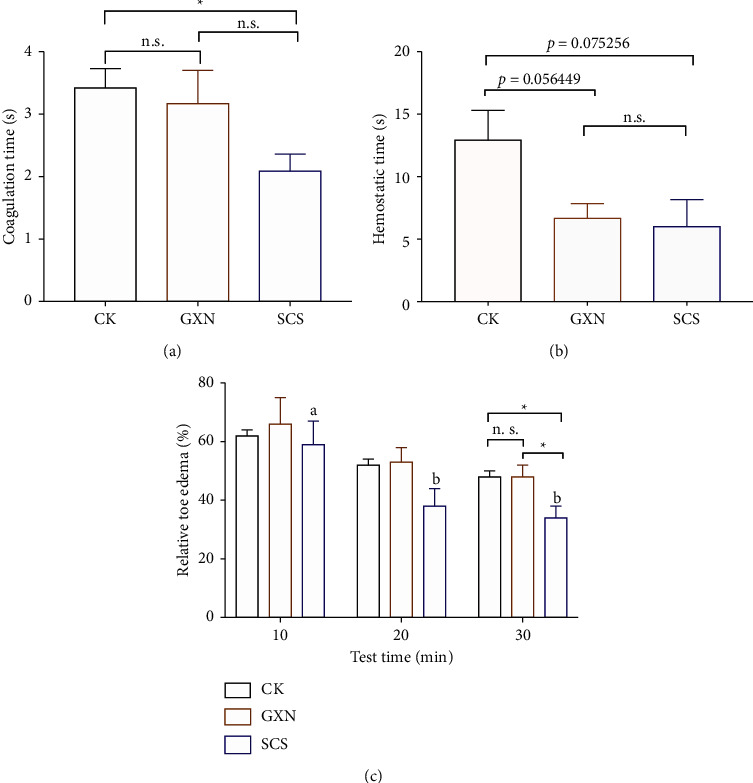
The effects of SCS drug on the coagulation, hemostasis, and anti-inflammation of SD rats. (a, b) Effects of different medical treatments on coagulation time and hemostatic time. (c) The changes of paw detumescence. Relative toe edema expressed by (toe volume after swelling-toe volume before swelling)/toe volume before swelling. Different superscripts (^ab^) or ^*∗*^asterisk (^*∗*^) indicates the significant difference in the different or same comparisons (*p* < 0.05) (*n* = 4).

Common drugs for the treatment of EH have the functions of hemostasis, anti-inflammation, and detumescence. Therefore, this study further compared the effects of SCS and GXN on hemostasis, anti-inflammation, and detumescence in the rats. Compared with CK and GXN groups, SCS harbored an accelerated blood coagulation effect on tail-broken rats (*p*=0.018) (Figure 4(a)), and similar alterations in the hemostatic time were also observed in the rats (Figure 4(b)). However, although both SCS and GXN had certain hemostatic functions, there was no significant difference between the two drugs. Compared with normal rats, the hemostatic time of both SCS (*p*=0.075) and GXN (*p*=0.056) was slightly decreased. Moreover, after injection with egg white saline, the relative toe edema in SCS rats reduced dramatically at 20 (*p*=0.012) and 30 (*p*=0.003) min comparing with 10 min, and the drug represented distinctive detumescence effect comparing with CK (*p*=0.021) and GXN (*p*=0.048) at 30 min (Figure 4(c)). Notably, the hematic phase results indicated that the content of erythrocyte (*p*=0.009), hemoglobin (*p*=0.011), and hematocrit (*p*=0.010) were decreased after SCS treatment, indicating that SCS drug could alleviate inflammation and swelling related to blood stasis (Table 1).

### 3.4. SCS Drug Restored Proliferating Endometrium

**Figure 5:**
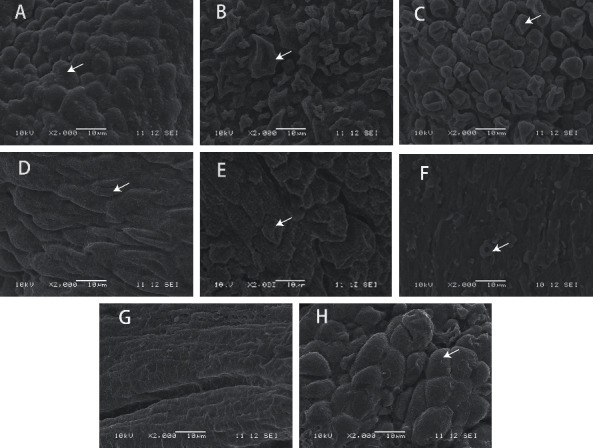
Scanning electron microscope (SEM) images of rat endometrium. (a) SEM image of CK rat endometrium. (b) SEM image of estradiol benzoate treated rat endometrium. (c–e) SEM images of rat endometrium after 3 d of gavage (c: EH, d: GXN, and e: SCS). (f–h) SEM images of rat endometrium after 7 d of gavage (f: EH, g: GXN, h: SCS). The white arrow indicates pinopode.

**Figure 6:**
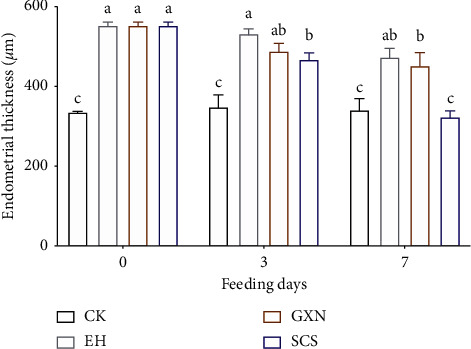
The effects of SCS drug on the thickness of endometrium in the endometrial hyperplasia rats. Values are means ± SEM. Different superscripts (^abc^) indicate the significant difference (*p* < 0.05, *n* = 4).

In the present work, we obtained EH rats to study SCS in the treatment of proliferating endometrium by injecting estradiol benzoate into rats for 60 consecutive days (Figure 5(b)). According to the scanning electron microscopy results, we found that most of the pinopodes (a morphological biomarker of endometrial epithelial cells) in the EH (Figures 5(c) and 5(f)) and GXN (Figures 5(d) and 5(g)) groups were atrophic, hollow, and sparsely arranged after 3 and 7 d of administration. In contrast, the pinopodes in the SCS cells gradually recovered to rounded after 7 d of administration (Figure 5(h)). After 3 d administration, the endometrial thickness in the SCS rats was dramatically reduced, and it was extremely different from the rats of other experimental groups on the 7 d (Figure 6). Importantly, the endometrial thickness gradually recovered to the state of CK, indicating that SCS could be used to treat proliferating endometrium.

### 3.5. SCS Altered HPO Axis-Related Hormone and Gene Expression

**Figure 7:**
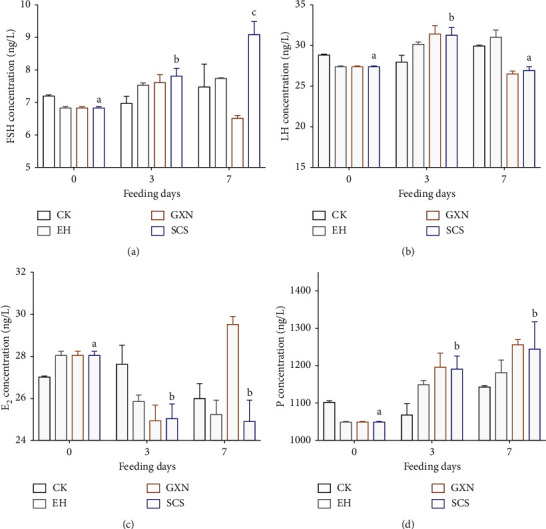
Effects of SCS drug on the hormone expression in the endometrial hyperplasia rats. The concentration of follicle-stimulating hormone (a), luteotropic hormone (b), estrogen (c), and progesterone (d) in the serum of rats. Values are means ± SEM. Different superscripts (^abc^) indicate the significant difference (*p* < 0.05, *n* = 4).

**Figure 8:**
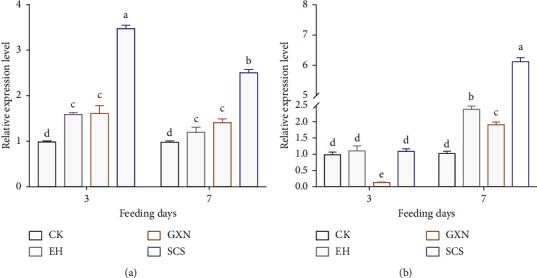
Effects of SCS on the MMP-1 and TIMP-1 expression in the endometrial hyperplasia rats. (a) mRNA expression levels of MMP-1 in endometrium; (b) mRNA expression levels of TIMP-1 in the endometrium. Values are means ± SEM. Different superscripts (^abcde^) indicate the significant difference (*p* < 0.05, *n* = 4).

To explore the molecular mechanism of SCS in the treatment of proliferating endometrium, we then detected four elemental hormones involved in the HPO axis. The results showed that FSH content in the SCS rats increased gradually with gavage time and was significantly higher than that in CK (*p*=0.083), EH (*p*=0.015), and GXN (*p* < 0.001) groups at day 7 (Figure 7(a)). Interestingly, both LH concentrations in GXN and SCS rats returned to normal after a short-term elevation (Figure 7(b)). Coupled with these are the decreased estrogen (Figure 7(c)) and increased progesterone (Figure 7(d)). In addition, SCS significantly increased the expression of the MMP-1 gene, and its significance was higher than that of CK (*p* < 0.001), EH (*p* < 0.001), and GXN (*p* < 0.001) groups at day 7 (Figure 8(a)). Similarly, the increased expression of the TIMP-1 gene in the SCS rats was observed, and the expression level was dramatically higher than that of CK (*p* < 0.001), EH (*p* < 0.001), and GXN (*p* < 0.001) groups at day 7 (Figure 8(b)). Two types of upregulated genes, MMPs and TIMPs, suggested that SCS could promote the degradation of hyperplasia endometrium by regulating the changes of HPO axis-related hormones.

## 4. Discussion

Considering the characteristics of TCM with low toxicity and difficulty in measuring lethal dose [27], the safety concerns such as treatment duration, long-term toxicity, and dose-dependent toxicity need to be meticulously examined in the use of TCM. For example, many species of *Aristolochia* in TCM were employed to treat symptoms such as acute arthritis and edema, but aristolochic acid extracted from the herb was found to contribute to renal impairment [28, 29]. Hence, before the drug is officially used, acute high-dose experiments need to be performed to detect the toxicity of SCS on rats. The results from the acute high-dose treatment elucidated that 20-dose SCS exerted no toxic effects on physiological parameters such as body weight in the rats. Furthermore, both ALT and AST are important biomarkers of liver function testing [30]. Still, the significance of the disease course and prognosis can only be determined when the ALT/AST ratio is changed [31]. Interestingly, liver histologic sections showed slightly increased gap junctions between hepatocytes in the SCS rats, indicating that the drug can elevate liver metabolic activity in the rats. Hence, the results from the blood biochemical parameters and histologic sections showed that high-dose SCS exerted no apparent impacts on the rat's liver. Also, the total protein decreased in the SCS rats, but the specific value was within the normal range [32]. In addition, the key component of total protein (i.e., albumin) showed no alterations compared with the control group [33, 34]. Nevertheless, it can not be omitted that SCS might induce slight liver toxicity, especially considering the used doses and duration of treatment, and further long-term assessment should be conducted to evaluate this issue. Also, we found that SCS exerted no significant impact on other organ indexes (kidney, spleen, and genitals). Thus, we assumed that SCS constitutes no obvious toxic effect on SD rats and could be used in subsequent tests. The normal dose SCS might also be used in clinical treatment of EH and relative complications, but future clinical tests would promise us a more convincing usage of medication.

DUB and proliferating endometrium can cause a range of bleeding symptoms, particularly persistent and uncontrolled uterine bleeding. Therefore, it is necessary to identify the hemostatic, procoagulant and antiphlogistic effects of the drug. The symptoms of hyperviscosity are characterized by increased hematocrit and red blood cell count [35]. Our results found that the value of erythrocyte, hemoglobin, and hematocrit decreased remarkably, indicating that SCS regulated blood substances and reduced blood viscosity in the rats, thus reducing the occurrence of blood stasis. This finding is consistent with previous studies showing that the rattans of *S. suberectus* are able to activate blood circulation and remove stasis [36]. In addition, SCS also shortened coagulation time and eliminated the relative toe edema in the rats. Interestingly, this observation agrees with previous studies that reported *S. chinensis* and *C. cristata* harbored efficient procoagulant and hemostatic effects [37, 38]. Hence, our results indicated that SCS could be served as an effective drug for complications caused by EH, such as bleeding, congestion, and inflammation.

Normal uterine bleeding is caused by periodic changes in the endometrium, regulated by the HPO axis. Morphological variations of the functional layer of endometrium can be divided into three phases: proliferative, secretory, and menstrual [39]. During the menstrual period, the levels of estrogen and progesterone dropped, which subsequently causes endometrial blood flow to decrease, resulting in endometrial necrosis due to ischemia. The area of damaged and ischemic necrotic tissue gradually expanded and the permeability of blood vessel wall boosted, eventually leading to tissue exfoliation [40, 41]. Anovulatory DUB results from the prolonged estrogen and limited progestin expression, which can trigger endometrium growth dramatically and cause breakthrough-estrogen-bleeding in females [42, 43]. Importantly, our findings demonstrated that pinopodes in EH rats gradually returned to functional morphology after SCS administration, showing that the drug had an inhibitory effect on EH and was more significant than the contrastive drug GXN. A similar study also reported that sauchinone, the active substance of *S. chinensis*, is able to inhibit the differentiation of bone-specific multinucleated cells by regulating the mitogen-activated protein kinases pathway [44]. Hence, we reason that SCS can prevent the occurrence of EH, thus lessening the bleeding caused by the endometrium shedding.

The gonadotropin-releasing hormone secreted by the hypothalamus can induce the synthesis of FSH and LH, thereby promoting follicular development and increasing estrogen secretion [45–47]. LH increases progesterone secretion and leads to ovulation. With the increase of estrogen and progesterone levels, the changes of hormones would produce negative feedback inhibition on the hypothalamus and pituitary gland, thus reducing the levels of FSH and LH, leading to luteal degradation [48–50]. Menstruation occurs when the endometrium loses these two hormones' support and exfoliates and bleeds. The imbalance of FSH and LH secretion affects the production of estrogen and progesterone and then induces the two-way feedback regulation of sex hormones, which causes female functional uterine bleeding [51, 52]. Estrogen can regulate uterine development and stimulate endometrium hyperplasia and thickening, whereas progesterone can stimulate endometrium proliferation under the stimulation of estrogen. The expression level of both increases simultaneously can cause endometrium thickening and functional uterine bleeding. Hence, we speculated that the inhibited hyperplasia of the endometrium was due to SCS regulating the related hormone expression levels in the rats. Test results echoed our opinion: after administration of SCS, the levels of FSH and progesterone in female rats increased, LH increased first and then decreased, and estrogen decreased. The increased FSH and LH could promote normal follicular development and menstruation. Importantly, the reduced estrogen content could inhibit the dysfunction of the endometrium, and the balanced estrogen and progesterone triggered healthy menstruation. This finding is in agreement with other studies that report that herbal formulas like B401 (a certified drug in Taiwan) can decrease endometrial thickness due to the increased content of estradiol-17*β* and the decreased expression of estrogen receptor [53]. Hence, our results showed the negative feedback mechanism of HPO, and the content of estrogen and progesterone did not elevate synergistically, thereby preventing the occurrence of EH.

MMPs are a group of proteases that degrade extracellular matrix (ECM), and TIMPs can specifically bind to MMPs to inhibit its activity and prevent further degradation of ECM [7, 54]. Studies have shown that the MMPs and TIMPs changed distinctively in ectopic endometrial patients [55]. The imbalance between MMPs and TIMPs promotes the degradation of ECM and increases the invasiveness of ectopic endometrium [56]. EH results from prolonged stimulation of the endometrium with unopposed estrogen, and many studies have shown that MMPs have significant differential expression in uterine fibroids, including high expression of MMP-1, MMP-2, and MMP-9 and low expression of MT1-MMP, TIMP-1, -2, and -3 [57–60]. Therefore, the increased MMP and TIMP levels are documented to balance the fibroid cell differentiation, which is in line with our findings. In the present study, the expression levels of MMP-1 and TIMP-1 were dramatically increased and highest among all groups, indicating that SCS could balance the expression of the MMP TIMP and genes, thereby promoting the degradation of ECM and reducing the EH. Taken together, to further validate this potential mechanism of regulation by MMP, TIMP, and female hormones, it would be necessary to compare the alterations of genes or intermediates (e.g., proteins, metabolites, transcriptional factors, and microRNAs) in both upstream and downstream of the EH-associated pathway and identify putative regulating factors. Such research will help evaluate and characterize the modulatory mechanism and specific function of SCS for the treatment of EH.

## 5. Conclusion

In conclusion, the present study proves the active components from three traditional Chinese herbs by TLC and HPLC methods. An acute high-dose test identifies the safety of SCS in the treatment of EH and DUB. This work provides evidence that alterations to the hormones and mRNA induced by the gavage of SCS could ameliorate EH of the rats. In addition, this drug is able to alleviate blood stasis and enhance coagulation and anti-inflammatory effects, which can be applied to treat complications of EH. Collectively, this integrated analysis generates a prospective strategy for the treatment of EH and DUB and plays a great role in the promotion of Chinese medicine.

## Figures and Tables

**Table 1:** Effects of SCS on the hematic phase in rats.

Test items	CK	GXN	SCS
Leukocyte (109/L)	24.70 ± 1.68	23.23 ± 2.42	14.27 ± 3.84
Lymphocyte (%)	57.30 ± 0.17	89.61 ± 2.23^*∗*^	63.60 ± 6.51
Monocyte (%)	5.43 ± 0.58	4.42 ± 1.29	5.40 ± 0.85
Neutrophil (%)	37.27 ± 0.44	23.32 ± 2.31^*∗*^	40.00 ± 7.01
Erythrocyte (1012/L)	8.16 ± 0.67	4.24 ± 2.13	3.67 ± 0.99^*∗*^
Hemoglobin (g/L)	174.67 ± 16.70	140.09 ± 21.03	77.67 ± 20.34^*∗*^
Hematocrit (%)	45.43 ± 4.17	26.89 ± 2.83^*∗*^	20.23 ± 5.32^*∗*^
MCV (fL)	55.63 ± 0.47	48.78 ± 2.78	56.20 ± 1.01
MCH (pg)	21.30 ± 0.35	19.27 ± 1.49	21.23 ± 0.96
MCHC (g/L)	384.00 ± 4.36	392.11 ± 3.42	383.33 ± 9.60
RDW (%)	10.27 ± 0.18	9.78 ± 1.21	12.57 ± 2.47
Platelet (109/L)	153.00 ± 23.30	222.31 ± 43.97	200.00 ± 68.60
MPV (fL)	6.13 ± 0.18	6.12 ± 0.78	6.80 ± 0.38
PDW (%)	17.23 ± 0.24	13.90 ± 2.11	18.50 ± 0.21^*∗*^
PCT (%)	0.26 ± 0.17	0.19 ± 0.08	0.14 ± 0.05

MCV: mean corpuscular volume; MCH: mean corpuscular hemoglobin; MCHC: mean corpuscular hemoglobin concentration; RDW: red cell distribution width; MPV: mean platelet volume; PDW: platelet distribution width; PCT: thrombocytocrit. The data in the table were mean ± SE, and asterisk (^*∗*^) indicated that there was a significant difference (*p* < 0.05) between CK and SCS/GXN group.

## Data Availability

The data used to support the findings of this study are available from the corresponding author upon request.

## Abbreviations

TCM: Traditional Chinese medicine
SCS: *Saururus chinensis*, *Celosia cristata*, and *Spatholobus suberectus*
TLC: Thin-layer chromatography
HPLC: High-performance liquid chromatography
HPO: Hypothalamus-pituitary-ovarian
DUB: Dysfunctional uterine bleeding
FSH: Follicle-stimulating hormone
LH: Luteinizing hormone
GXN: Gongxuening capsule
CK: Blank control group
EH: Endometrial hyperplasia
SEM: Scanning electron microscopy
MMP-1: Matrix metalloproteinase-1
TIMP-1: Tissue inhibitor of matrix metalloproteinase-1
TP: Total protein
ALT: Alanine aminotransferase
AST: Aspartate aminotransferase.
